# ECG Monitoring Systems: Review, Architecture, Processes, and Key Challenges

**DOI:** 10.3390/s20061796

**Published:** 2020-03-24

**Authors:** Mohamed Adel Serhani, Hadeel T. El Kassabi, Heba Ismail, Alramzana Nujum Navaz

**Affiliations:** 1 Department of Information Systems and Security, College of Information Technology, UAE University, Al Ain 15551, United Arab Emirates; 201570182@uaeu.ac.ae; 2 Department of Computer Science and Software Engineering, College of Information Technology, UAE University, Al Ain 15551, United Arab Emirates; htalaat@uaeu.ac.ae (H.T.E.K.);

**Keywords:** ECG, ECG monitoring system, smart monitoring, heart diseases, cardiovascular diseases, IoT, sensors

## Abstract

Health monitoring and its related technologies is an attractive research area. The electrocardiogram (ECG) has always been a popular measurement scheme to assess and diagnose cardiovascular diseases (CVDs). The number of ECG monitoring systems in the literature is expanding exponentially. Hence, it is very hard for researchers and healthcare experts to choose, compare, and evaluate systems that serve their needs and fulfill the monitoring requirements. This accentuates the need for a verified reference guiding the design, classification, and analysis of ECG monitoring systems, serving both researchers and professionals in the field. In this paper, we propose a comprehensive, expert-verified taxonomy of ECG monitoring systems and conduct an extensive, systematic review of the literature. This provides evidence-based support for critically understanding ECG monitoring systems’ components, contexts, features, and challenges. Hence, a generic architectural model for ECG monitoring systems is proposed, an extensive analysis of ECG monitoring systems’ value chain is conducted, and a thorough review of the relevant literature, classified against the experts’ taxonomy, is presented, highlighting challenges and current trends. Finally, we identify key challenges and emphasize the importance of smart monitoring systems that leverage new technologies, including deep learning, artificial intelligence (AI), Big Data and Internet of Things (IoT), to provide efficient, cost-aware, and fully connected monitoring systems.

## 1. Introduction

The last decade has witnessed an increasing number of deaths caused by chronic and cardiovascular diseases (CVDs) in all countries across the world. CVDs are disorders affecting the blood vessels and the heart. CVDs involving the blood vessels are known as vascular diseases, such as coronary artery disease. Those involving the heart include heart failure, cardiomyopathy, rheumatic heart diseases, stroke, heart attack, and arrhythmias. 

According to the World Health Organization (WHO), CVDs are the number one cause of death globally, with 17.9 million deaths every year [1]. It remains the number one cause of death of all Americans, claiming more than 840,000 lives in 2016 [2]. Furthermore, the European Health Network European Cardiovascular Disease Statistics 2017 edition revealed that CVDs cause 3.9 million deaths in Europe and over 1.8 million deaths in the European Union (EU) yearly. This accounts for 45% of all deaths in Europe and 37% of all deaths in the EU [3].

Continuous heart rate monitoring and immediate heartbeat detection are primary concerns in contemporary healthcare. Experimental evidence has shown that many of the CVDs could be better diagnosed, controlled, and prevented through continuous monitoring, as well as analysis of electrocardiogram (ECG) signals [4,5,6,7,8,9]. Hence, the monitoring of physiological signals, such as electrocardiogram (ECG) signals, offers a new holistic paradigm for the assessment of CVDs, supporting disease control and prevention. With advances in sensor technology, communication infrastructure, data processing, and modeling as well as analytics algorithms the risk of impairments could be better addressed more than ever done before. This, in turn, would introduce a new era of smart, proactive healthcare especially with the great challenge of limited medical resources.

As a result, ECG monitoring systems have been developed and widely used in the healthcare sector for the past few decades and have significantly evolved over time due to the emergence of smart enabling technologies [10,11,12,13]. Nowadays, ECG monitoring systems are used in hospitals [14,15,16,17], homes [18,19,20], outpatient ambulatory settings [21,22,23], and in remote contexts [24]. They also employ a wide range of technologies such as IoT [25,26,27], edge computing [28,29], and mobile computing [30,31,32]. In addition, they implement various computational settings in terms of processing frequencies, as well as monitoring schemes. They have also evolved to serve purposes and targets other than disease diagnosis and control, including daily activities [33,34,35], sports [36,37,38], and even mode-related purposes [39,40,41].

This massive diversity in ECG monitoring systems’ contexts, technologies, computational schemes, and purposes makes it hard for researchers and professionals to design, classify, and analyze ECG monitoring systems. Some efforts attempted to provide a common understanding of ECG monitoring systems’ processes [42,43,44,45,46,47], guiding the design of efficient monitoring systems. However, these studies lack comprehensiveness and completeness. They work for specific contexts, serve specific targets, or are suitable for specific technologies. This makes the available ECG monitoring system processes and architectures hard to generalize and reuse. On the other hand, some studies attempted to analyze ECG monitoring systems’ attributes and provide classification taxonomies, supporting better analysis and understanding of the ECG systems reported in the literature. However, exiting reviews related to ECG monitoring in the literature can be intuitive and incomprehensive [48]. They do not consider the latest technological trends [49,50,51], and they target very narrow research niches, such as wearable sensors [52,53,54,55], mobile sensors [56], disease diagnosis [57], heartbeat detection [58], emotion recognition [59], or ECG compression methods [60]. Hence, there is a need to provide a comprehensive, expert-verified taxonomy of ECG monitoring systems, a common architecture, and a complete set of processes to guide the classification, analysis, and design of these systems.

Therefore, in this work, we propose an expert-verified taxonomy of ECG monitoring systems, a generic architectural model, and a complete, general set of processes to support better understanding, analysis, design, and validation of ECG monitoring systems from a broader perspective. Our experts’ taxonomy is composed of five distinct, cohesive clusters. Each cluster focuses on one dimension of ECG monitoring systems, detailing the features and attributes of these systems in that dimension. These include monitoring contexts, technologies, schemes, targets, and futuristic monitoring systems. In addition to our experts’ taxonomy, the proposed ECG monitoring systems’ layered architecture depicts essential structural components and elements of ECG monitoring systems, their interfaces, and the data inputs/outputs of each layer. We also complement our experts’ taxonomy and the generic architecture with a comprehensive ECG monitoring process model, highlighting the major processes, sub-processes, and supporting processes, emphasizing factors adding value to each process. Based on the proposed taxonomy, architecture, and common process model, we conduct an extensive, thorough analysis of the literature surrounding ECG monitoring systems, highlighting systems’ categories, attributes, functions, challenges, and current trends, leading to a panorama of ECG monitoring systems. To our best knowledge, this is the most comprehensive, expert-verified review of ECG monitoring systems to date.

This paper is organized as follows: Section 2 introduces the ECG monitoring architecture, describes the monitoring value chain and the main processes involved. Section 3 describes the experts’ taxonomy of ECG monitoring systems. It details, compares, and analyzes each cluster of studies, as well as highlights the key features and reviews some research challenges. Section 4 emphasizes the key challenges related to ECG monitoring systems. Finally, the last section summarizes and discusses our findings and points to future research directions for ECG monitoring systems.

## 2. Overview of ECG Monitoring Systems: Architecture, Processes, and Technologies

### 2.1. ECG Monitoring Architecture

**Figure 1:**
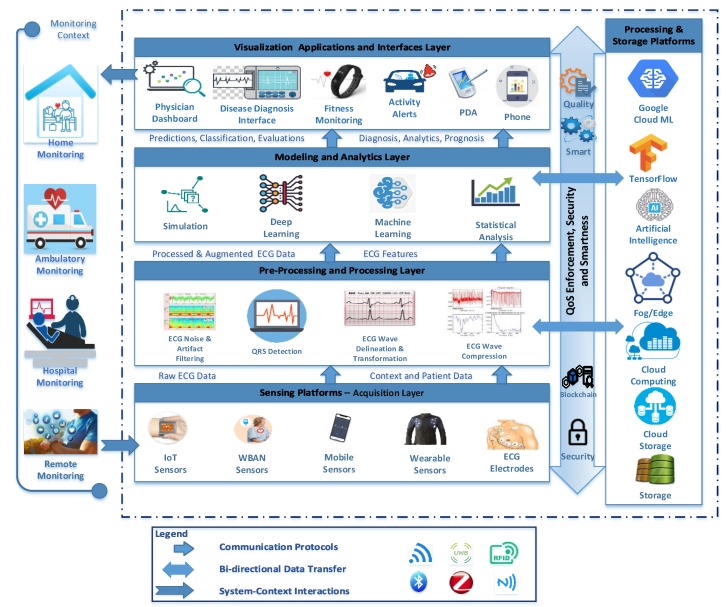
The overall architecture of electrocardiogram (ECG) monitoring systems.

Figure 1 describes a generic ECG layered monitoring architecture that we have developed to capture the key elements and structural components of ECG monitoring systems. These include the main processes involved, the underlying platforms, and the main actors and their involvement in the ECG-based monitoring processes. The architecture is organized into four horizontal connected layers and one vertical silo on the right in addition to important properties that have to be supported across all layers including security fulfillment, Quality of Service (QoS) enforcement, and smartness integration. This architecture delivers the service through different monitoring contexts, as depicted on the left side of the figure. In fact, the monitoring context represents the environment where the ECG monitoring system is deployed and the monitoring activities take place. Our proposed architecture is designed to fit into contexts of use in which various actors interact with the ECG monitoring system by providing inputs and receiving some sort of output. Hence, we dissociated the contexts of use and possible interactions of various actors within these contexts from the monitoring system.

The monitoring context can vary from home monitoring, to ambulatory monitoring, to hospital monitoring, to remote monitoring. The data comes from the context into input layer, moves through the system, then goes back to the context through the visualization layer. This gives a complete realization of the system within its contexts. The architecture encompasses four layers: the bottom layer is the acquisition layer, which offers various sensing platforms and devices, such as ECG sensors, IoT sensors, Wireless Body Area Network (WBAN) sensors, mobile sensors, and wearable sensors. Sensors vary from embedded sensors mounted on biological tissues to comfortable, easy-to-handle smart watches and smart vests. Cardiac data in the form of ECG signals along with context and patient data are acquired using several communication protocols autonomously using Wifi, Bluetooth, ultra-wide band (UWB), ZigBee, radio-frequency identification (RFID), and near-field communication (NFC) and then transported to the next layer, which is the preprocessing and processing layer. The latter is responsible for handling operations such as ECG noise and artifact filtering, QRS detection, ECG wave delineation, transformation, and compression. Processed and augmented ECG data and features are then relayed using the aforementioned communication protocols to the modeling and analytics layer, where different machine learning, deep learning, and statistical analyses are conducted to analyze, extract patterns from ECG data, and identify and predict various heart diseases, such as arrhythmia, atrial fibrillation, epilepsy, mental fatigue, and fainting. The outputs generated from this layer are then delivered to the visualization layer where different applications and device interfaces (e.g., physician dashboard, disease diagnosis interface, and patient mobile device) are used to visualize analyzed data which can be effectively used in disease diagnosis, physician feedback, activity monitoring and alerts to drivers, fitness and general monitoring.

The right silo provides processing and storage services to all processes in the four horizontal layers of the architecture. For example, processed real-time ECG signals might utilize Fog/Edge computing. Also, Cloud infrastructure offers storage and processing services at various stages of the ECG monitoring lifecycle. The TensorFlow platform provides an execution environment, offering a multitude of deep learning libraries, packages, and visualization tools to test various deep learning models.

At the core of this ECG monitoring architecture, security and data privacy are important characteristics that should be supported in all processes where data are collected, transferred, processed, analyzed, accessed, and visualized by various stakeholders. Blockchain technology can be integrated to provide a trusted, decentralized, and immutable ledger for various transactions which outperforms existing methods, techniques, and mechanisms. It provides a high level of transparency to ensure security and privacy. It can, for example, integrate IoT devices and regulate the IoT device’s behavior automatically. All activities in the IoT and related transactions are recorded into blockchain through smart contracts for secure data logging and auditing at a low cost. QoS is also a very important system’s characteristic that needs to be supported at every single process, including, for instance, ensuring the quality of the data, the quality of the preprocessing and processing, the quality of analytics results, and the quality of visualization. Quality measurement and enforcement are supported through continuous monitoring and control of various proprieties of the monitoring system. Monitoring logs are continuously analyzed and improvement measures are taken to react to any quality degradation. Finally, smartness is another property of the ECG monitoring systems where various intelligent features could be implemented across all layers from data inception to visualization. Such intelligent features encompass instrumenting sensors to preprocess the data at the sensor or at the edge, energy-harvesting of sensors and mobile devices, self-adaptation, and self-learning algorithms that react to dynamic environment changes to make some intelligent decisions.

### 2.2. ECG Monitoring Value Chain: Comparative Study

ECG monitoring value chain encompasses a set of common processes, including data acquisition, preprocessing, feature extraction, processing, analysis, and visualization. Studying and analyzing the value chain of ECG monitoring systems helped in understanding the value and contribution of each process within the system, the best practices that can be adopted within each process, and the ultimate goal of the overall system in guaranteeing higher quality disease diagnosis and increased resource utilization efficiency in terms of energy and cost. Most of the existing researches agreed on the main ECG monitoring processes mentioned above. However, depending on the nature of the monitoring application, some research work has defined additional distinct or overlapping processes, such as data cleansing, encryption, and compression, but could be incorporated or merged as part of existing primary processes, or isolated as supporting processes.

We intend to identify research gaps in defining the complete lifecycle of ECG monitoring systems and highlight the existing models, which include processes that overlap, or processes that are merged. Our main objective is to highlight the added value these processes provide to the monitoring system’s lifecycle, as well as possibilities for optimization and improvement. One of the most important processes is data acquisition;it involves measuring and recording the heart’s activity using different sensors. The massive data generated by the ECG acquisition process requires preprocessing to prepare the data for the next stages, which are feature extraction and processing. The accuracy of preprocessing indirectly affects the subsequent stage of the value chain. Such preprocessing activities include cleansing the ECG data without losing its main components and features. This is why most of the research work devotes huge efforts to the preprocessing stage. Having huge volumes of ECG signals necessitates the feature extraction process to reduce the amount of processing and save resources prior to the processing and analysis stage. Features extraction is a very critical stage, as it has a significant effect on the subsequent stages of the lifecycle. The processing and analysis stage requires the application of various optimization techniques to achieve higher accuracy, precision and quality results. This is the most important stage in the monitoring system’s lifecycle as it affects the accuracy of signal interpretation and diagnostics. Finally, the different visualization tools enable end-users to clearly and efficiently visualize the results of the monitoring systems. This stage is also significant as it allows accessibility, usability, and understandability of complex data.

A review of the literature on ECG monitoring systems has differentially defined the processes/stages of the ECG monitoring system’s lifecycle, as depicted in Table 1. In this table, the value chain throughout various lifecycle representations of existing ECG monitoring systems is given emphasis. None of the proposed initiatives provides a full description of the complete lifecycle of an ECG monitoring system, including both the primary and the supporting processes. Most of these initiatives barely cover 70% of the primary processes throughout the lifecycle. Supporting processes were described in nearly half of the proposed work included in Table 1, such as data storage and data modeling.

The most inclusive ECG monitoring system’s lifecycles that included the primary processes definition were depicted in [55,58,72,75,78,83]. Few of the researches specified additional supporting processes, such as data storage or encryption. Typically, IoT-based monitoring systems adopt most of the primary processes [12,13,71,72]. Other studies emphasized only a partial lifecycle that has to do with data acquisition, preprocessing, and processing for heartbeat detection or finding an annotation set depending on the purpose of the system [43,44,45,49,63,65]. However, few initiatives focused only on one process, such as processing in [64].

Supporting processes, such as training and modeling, were highlighted to support machine learning and neural network techniques [68,73]. Data cleansing was also defined as a discrete process in [71]. Additional important supporting processes were introduced in [72] to enforce privacy and security; these are responsible for encryption and decryption.

### 2.3. The ECG Monitoring Key Processes

**Figure 2:**
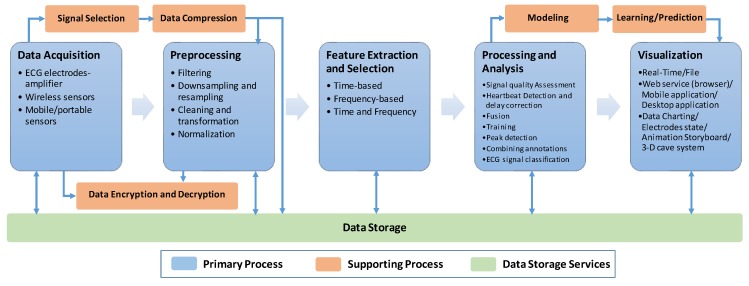
ECG Monitoring Lifecycle.

There is no clear and comprehensive definition of the ECG monitoring system’s lifecycle processes in the literature. Most researchers focus on a subset of key processes; however, they neglect other very important supporting processes. Therefore, we will provide an across-the-board description and classification of primary and supporting processes that should be implemented within an ECG monitoring system, as depicted in Figure 2.

The following subsections describe the details of each primary process within the generalized lifecycle of an ECG monitoring system. We intend to eliminate the overlapping and process definition ambiguities and highlight the adaptability of the processes to the unique needs of each system.

#### 2.3.1. ECG Data Extraction and Collection

An ECG is considered the best method for detecting heart abnormalities. The available ECGs vary from single to 12-lead ECG recording devices. On one hand, hospital ECG acquisition devices are usually big in size and support high-precision and long-term monitoring. However, they restrain the patients’ movements and involvement. On the other hand, wearable health-monitoring systems afford real-time continuous monitoring of patients’ through the deployment of multiple sensors. 

The process of data acquisition, the first stage/process in the lifecycle of ECG monitoring systems, encompasses the selection of the following: (1) the types of sensors (e.g., wireless, or wired), (2) the placement location of sensors, (3) the number of sensors, and (4) the hardware required for data acquisition, storage, and transmission [51]. Nevertheless, in some ECG monitoring systems, real-time and continuous ECG sensor acquisition is handled. ECG signal acquisition is a challenging task because of its sensitivity towards various quality dimensions including precision, accuracy, and timeliness. Inaccurate data collection may lead to a wrong diagnosis and accordingly affect clinical decisions.

However, most researchers working with ECG monitoring systems favor datasets from well-known databases, rather than creating a data acquisition system of their own, especially when they address diagnosis issues and feature extraction techniques, which constitute the remaining parts of the monitoring lifecycle. A thorough review of existing databases that provide single-lead and multi-lead ECG signals was depicted in [58].

#### 2.3.2. Preprocessing

Preprocessing is intended to enhance the accuracy of prediction; it improves the quality of the raw signal, removes the noise, and removes the baseline wander and powerline interference. The noise is classified, in the literature, into five main groups: powerline interference, baseline wander, electrode contact noise, electrode motion artifacts, and muscle contractions. The most common preprocessing and noise removal techniques are classified into three main categories: Wavelet Transform-based, curvelet transform-based, and adaptive digital filters [86].

The preprocessing of ECG signals related to the cardiac cycle, such as duration of the QRS complex and the ST–T segment level, is better handled using linear filters to prevent phase distortion from fluctuating wave properties [84]. However, reducing the effect of noise caused by muscle activity requires averaging techniques to the time-aligned heartbeats. Existing standard filtering techniques lack efficiency in handling the signals. Therefore, hybrid filtering is rather more adaptive to raw ECG signals and, thus, was introduced in some research work to improve filtering results [89,90].

Despite being challenging for preserving important signal information, and adapting to the patient’s features, preprocessing has attracted the attention of researchers. Cleaning and transformation are also performed during the preprocessing stage [91]. Other techniques are also used during the preprocessing stage, such as downsampling, resampling and signal normalization [58].

#### 2.3.3. Feature Extraction

One of the most important processes in the ECG monitoring lifecycle is the feature extraction process. It contributes significantly to cardiac disease diagnoses, as it retrieves the most representative set of features from the preprocessed ECG, which allows better heartbeat detection. The features comprise a collection of summarized signal information used to characterize patterns. These features are extracted using different computation algorithms and techniques to help researchers, along with the support of visual skills expertise in problem diagnostics.

The features of ECG signals include, but are not limited to, the area under the curve, peak amplitude, time delay between peaks and valleys, and heart rate frequency. The main feature extraction methods are Wavelet Transform-based feature extraction, the autocorrelation function-based feature extraction method (periodic information of ECG signals), principal component analysis-based feature extraction method (finding periodic information in time series signals), and normal feature extraction method (Fast Fourier Transform (FFT)) [92]. Tejedor et al. [58] classified feature extraction techniques into three approaches based on the type of features extracted, these are: (1) time-based, such as the maximum amplitude pair function and the symbolic discretization method from a time series, (2) frequency-based, such as Fast Fourier Transform, power spectral density, and Gaussian and moving average low-pass filters, and (3) a mix of both time and frequency, such as Wavelet Transform, frequency QRS power, and maximum and minimum amplitudes used for the detection of heartbeats. 

Finding the optimal set of features that capture the true nature of the ECG signals remains a challenging task. Researches tried to apply multiple techniques to address the aforementioned issue, such as adopting Discrete Wavelet Transform and the Pan Tompkins Method to improve heartbeat abnormality classification from ECG signals in [93], or using time domain, frequency domain and distribution features for detection of atrial fibrillation (AF) in [46]. Keeping the good reliability of data and the quality of the signal are also challenges facing smartphone-integrated ECG monitoring systems; such a case was handled in [94] by enhancing the feature extraction process. The Wavelet Transform was also used in [7,60,67,95,96]. Alternatively, Wavelet Transform, combined with other techniques, was also applied in [7,66,97].

#### 2.3.4. Processing and Analysis

Intensive research was devoted to improve the efficiency of processing and analysis of ECG signals to achieve high diagnostic accuracy. During the processing phase, advanced information technologies are carried out through the development of diverse algorithms and intelligent techniques, such as analysis, modification, and synthesis applied to ECG signals to recognize and identify its significant components, with the purpose of discovering diagnostic information. These include, but are not limited to signal quality assessment, ECG signal classification, heartbeat detection and delay correction, peak detection, and training. Processing ECG signals is challenging due to their special characteristics, such as dynamicity, noise vulnerability, and inconsistency among individuals. Therefore, the optimization and development of ECG signal processing techniques has attracted research interest.

AI methods and neural networks are typically very useful in providing ECG signal interpretation. In the following, we depict a few examples from the literature of different techniques used for ECG signal processing [68,98,99,100,101]. Recent research in the literature adopts Neural Network (NN) and decision trees for the diagnosis of different cardiac diseases, the assessment of cardiac health conditions, the detection of chronic problems, sleeping issues including apnea, and mood and emotion recognition [55]. Other examples include using deep learning for the automatic recognition of ECG signals [102], a convolutional neural network (CNN) for arrhythmia classification in [103] and AF in [104], and recurrent neural network (RNN) for activity prediction [105]. Different machine learning algorithms have been applied in IoT Cloud-based monitoring systems in [13] and Cloud-based cardiology services in [106].

#### 2.3.5. Visualization

The visualization process typically includes all the functionalities that will allow users to inspect and interact with recorded or annotated ECG signals in real-time, as well as offline from a file [80,81]. This process acquires its importance throughout the value chain as it helps the human brain to better understand and analyze patterns, and detect abnormalities, especially with large datasets. The visualization applications vary in terms of the hosting application, nature of information projected, and the functionalities supported, which can be implemented as a web service through a browser, mobile applications [71], or desktop applications [107]. There are many commercial applications for ECG monitoring in the market, such as Custo Med, Philips, NORAV, and MEDSET [108]. 

Motalova et al. [82] proposed a design for web application visualization to display data from the ECG device. The application included data charting, electrode state, and animation storyboard functionalities. A three-dimensional cave interactive system was proposed in [81] which provides a graphic user interface that demonstrates a three-dimensional modeling and animation of a human heart using the R-wave of the electrocardiographic signal. 

#### 2.3.6. Supporting Processes

The supporting processes are the activities that provide extra functionalities to support the primary processes to realize an efficient monitoring system. In other words, they are not compulsory for every ECG monitoring system. For example, the signal selection process is performed only in systems using multiple physiological types of signals for heartbeat detection [58,70] or R-peak detection [62]. Additionally, the data compression process is required for many purposes, including storage capacity reduction and faster file transfer, which eventually contributes to efficient bandwidth utilization and cost reduction, especially in the case of continuous ECG monitoring and data streaming. Various data compression techniques were proposed in [84,85,88]. Němcová et al. [79] presented a thorough literature survey on data compression methods, as well as quality assessment techniques applied after compression.

Another example of a supporting process, as depicted in Figure 2, is data encryption and decryption. This process plays an important role in supporting security and data privacy, which are incorporated in very few ECG monitoring systems. Page et al. [72] used a fully homomorphic encryption (FHE) technique for the data encryption in a remote monitoring system.

The modeling and learning processes are considered the most important supporting processes required for machine learning, and prediction or prognosis of cardiovascular diseases. Although they are defined as the main processes for prediction-based systems, not all ECG monitoring systems support prediction. Therefore, we consider it as one of the supporting processes, rather than a primary process. Numerous monitoring systems define modeling, learning and prediction [75]. Some research proposals define a distinct training process [35,68] deep learning (ANN) process [83,87], machine learning (ML) process [86], and ML streaming process [13].

Nevertheless, data storage is considered a supporting process because it services many of the primary processes along the lifecycle of ECG monitoring systems. The data storage process is defined as a standalone process in [13,71,76], and for encrypted data storage in [72].

## 3. Experts’ Taxonomy of ECG Monitoring Systems

Due to the continuous expansion in the number of ECG monitoring systems proposed in the literature, it is very hard for researchers in this field to analyze and classify these systems into distinct, cohesive clusters of interrelated works. In this research, we use the term “cluster” to refer to a category of research works sharing common characteristics, attributes, and features. We use these clusters to classify papers related to ECG monitoring systems. The authors of this work have adopted a systematic approach that starts with (1) collecting resources (e.g., journal articles, conference papers, and book chapters) from various databases including Scopus, IEEE, Springer, and ACM, (2) conducting numerous preprocessing activities which involve, for instance, cleaning, reorganizing, removing duplications, and deleting non-system based solutions, (3) running extensive meeting sessions to classify and group these systems based on the surveys conducted in more than 600 publications, (4) sharing the clustering results with both medical and technological experts in the field to seek their feedback and inputs, and (5) finally, meeting with all involved parties to finalize and refine the final clusters of ECG monitoring systems.

**Figure 3:**
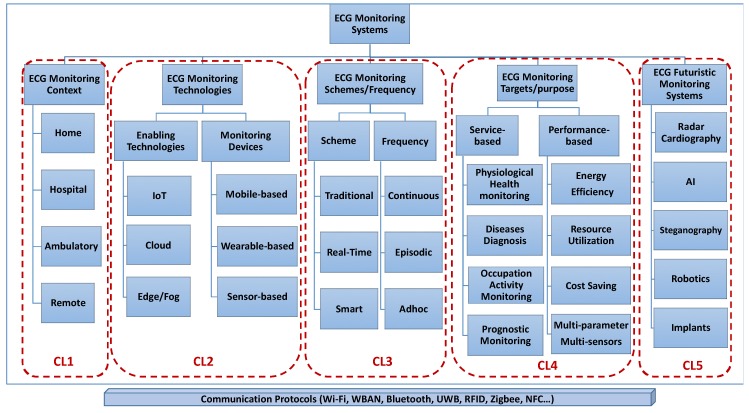
ECG monitoring system clustering.

Figure 3 presents ECG monitoring systems divided into four main clusters, in addition to the fifth cluster, which is considered the future generation of ECG monitoring systems. These are clustered based on the monitoring context for which they are developed, the involved ECG technologies and devices that characterize these systems, the monitoring scheme and frequency adopted, the monitoring target and purpose for which these systems were implemented, and finally, the futuristic monitoring system that leverages new technologies, such as AI, robotics, and nanotechnology, to shape emerging ECG monitoring systems. In addition to the five clusters, a horizontal underneath level represents the communication protocol and the wireless technology used by all the categories of monitoring systems to transport the ECG signals from sensors to the underlying servers and/or devices under which the ECG is processed and visualized.

In the following sub-sections, we describe and analyze each of the abovementioned clusters and we provide a comprehensive review of each cluster in terms of integrated ECG monitoring systems and solutions.

### 3.1. Context-Aware ECG Monitoring Systems

The first cluster of work incorporates the group of systems that are organized into monitoring contexts for which ECG monitoring systems were developed and consist of home-based, hospital-based, ambulatory-based, and remote-based ECG monitoring systems. Within each of these environments, commonalities exist and variations are also evident.

In Table 2, we describe the selected context-aware ECG monitoring systems and their categories, along with the selected papers under each category.

#### 3.1.1. Home ECG Monitoring Systems 

ECG Monitoring systems deployed in the home environment are generally classified into what is called telemonitoring, wearable continuous monitoring, and monitoring the elderly people in their homes. These systems were developed to reduce the economic burden on hospitals and involve patients in their continuous health monitoring with the comfort of being at home and are designed for people with lifelong and chronic disease or elderly people that require permanent assistance, surveillance and monitoring.

Telemonitoring is one of these systems which is integrated mostly in home or hospice settings to enable nurses to efficiently monitor patients, react to problems, and treat them before they propagate to more serious issues. Many researches proposed telemonitoring systems [109,110,111,112]; an example of these systems includes the work of [109], which uses mobile phones and NFC equipment to support AF monitoring. System feasibility, usability, and patient devotion have been tested in a clinical environment. Another body of work in [110] proposed the use of mobile Cloud computing to overcome issues related to the computational requirement of the large amount of data processing resulting from continuous ECG monitoring. Coronary heart disease risk is identified using feature extraction and an adaptive neuro-fuzzy inference system-based classification has been used. Similarly, in the work of Wang et al. [111] a hybrid mobile-Cloud ECG telemonitoring method has been proposed to allow more effective personalized medical monitoring and convey processing to the Cloud whenever heavy processing is required and cannot be handled by mobile devices. Likewise, Benini et al. [112] proposed a user-friendly single-lead ECG device designed for patients’ use at home. The device requires few calibrations to be operated and it is able to send, transparently, the acquired ECG to the surveillance center through a Bluetooth connection and via an Internet gateway.

The second category of home-based ECG monitoring systems involves wearable continuous monitoring, among which are the following propositions [18,19,20,128]. While according to the authors of [18], the effect of home-based wearable continuous ECG monitoring covers the discovery of undiagnosed AF in a randomized clinical trial, in [20] a real-time wearable ECG monitoring system for cardiac arrhythmia classification is introduced in a living environment. A review paper by the authors of [19] focuses on remote monitoring of cardiac functions, overviews the existing ECG data storage format standards and identifies their limitations.

The third category of home-based ECG monitoring involves elderly monitoring; several systems have been developed, among which include [24,113,114,115]. Mena et al. [115] proposed a mobile personal elderly health monitoring for automated classification of ECG signals using machine learning techniques. A wearable ECG monitor is integrated with a self-designed wireless sensor for ECG signal acquisition and is used with a native, purposely designed smartphone application. Other alternative studies [24,113,114] introduced various smart features to ECG monitoring systems for the elderly people. Smartness is introduced in the elderly monitoring environment where a novel monitoring framework is proposed in [113] to provide flexibility and enable interoperability between a myriad of healthcare monitoring devices. It relies on the analytics of evidence-based data collected from sensors, as well as the massive data collected from social networks. Smartness was also implemented in [114] through smart clothes for elderly ECG monitoring, which consisted of conductive fiber clothes to acquire ECG, a gateway to relay the data, and a server to process the data.

#### 3.1.2. Hospital ECG Monitoring Systems

Hospital-based ECG monitoring systems are classified into systems developed either for an intensive care unit (ICU) clinical setting [16,17], non-ICU clinical setting [14,15,116,117], or a Holter monitoring setting [36,118,119,120,121]. ECG Monitoring systems for ICUs involve, for instance, the work depicted in [16], which used data mining techniques to predict mortality and length of stay in an ICU. The authors presented an analysis of a mortality prediction algorithm to evaluate the extent to which the proposed algorithm can predict mortality rate. Similarly, Ahouandjinou et al. [17] proposed a hybrid architecture to visually monitor the patient for automatic detection of risk situations and alert generation using a multi-camera system and collaborative medical sensors network.

ECG monitoring systems for non-ICUs involve textile-based, contactless ECG monitoring, as proposed in [14]. This consists of monitoring contactless the vital signs with capacity sensors embedded in various patients’ auxiliaries. Also, a monitoring device has been developed for remote monitoring of out-of-hospital patients’ heart conditions in [15]. It consists of a real and flexible collection of ECG data from patients, obtaining valuable information that can be used for remote research and diagnosis. Finally, best practices for hospital ECG monitoring have been proposed in [116] and a set of recommendations are made, which included indications, timeframes, and strategies to improve the diagnostic accuracy of cardiac arrhythmia, ischemia, and QT interval monitoring.

Holter-based ECG monitoring is another type of system proposed in [36,118,119,120,121]; it is connected to the patient’s body through electrodes. In [118], a small and smart ECG Holter monitoring system integrated smartphones to retrieve ECGs from sensors, classify and detect abnormal signs. Other studies [119,121] used a Holter monitoring system to detect and improve AF in stroke patients. This is a very challenging research problem, as episodes are often short, occur randomly, and are frequently asymptomatic. The specific use of a Holter monitor can be found in [36] to record ECG underwater. ECG was analyzed for heart rate, arrhythmias, conduction abnormalities and ischaemic events in relation to various diving stages.

#### 3.1.3. Ambulatory ECG Monitoring Systems 

Considerable research and development is being undertaken for ambulatory ECG monitoring systems [8,21,22,23,122,123,124,125]; most of the researches support data collection, transmission, and analytics for ambulatory emergency situations. An example is the project depicted in [123], which implements a low-cost, high-efficiency and effective wireless real-time system for health monitoring through a telemetry system for on-spot-accident patients. The authors of [124] developed a wireless capacitive sensor for ambulatory ECG monitoring over clothes, while the authors of [21] proposed a modified Lewis ECG lead system for the ambulatory monitoring of atrial arrhythmias.. Finally, compressed sensing has been introduced in [23] for real-time energy-efficient ECG compression in a Wireless Body Sensor Network (WBSN).

Several research works on wearable ECG monitoring have been developed in the literature [25,29,125,126,127,128]. These works can be classified into textile-based systems, such as [29,128], and contactless based systems, such as [126]. Textile-based solutions integrate clothing (e.g., a shirt) with integrated sensors recording ECG and an acquisition module for data storage and processing. However, contactless-based monitoring systems are non-invasive and do not require direct body contact to retrieve ECG and other vital signs. Both categories of systems have been implemented for monitoring, for example, heartbeats, to detect patterns that might point to arrhythmia in diverse contexts, such as ambulatory and home settings.

#### 3.1.4. Remote ECG Monitoring Systems 

The telemonitoring method proposed within the context of remote ECG monitoring system in [10,129,130] differs from the one described for the home monitoring context above. It is designed and developed for distant patients whose movement is very frequent (e.g. exercising, doing sporting activities, and/or working), which does not necessary require his/her presence at home. Such systems include a remote health monitoring system for detecting varying cardiac disorders, including, for instance, arrhythmia and myocardial conditions, as proposed in [129]. Likewise, Tewary et al. [130] designed and developed a global system for a mobile-based smart wearable system capable of detecting sudden fall situations, cardiac abnormalities, and hypertension/hypotension; thus making it suitable for real-time monitoring, self-diagnosis, and remote diagnosis purposes.

Numerous research initiatives developed smart devices for ECG monitoring [12,24,126,131,132], where these devices implemented intelligence either in the sensors themselves or within the network components. Examples of these researches include a remote patient monitoring system for cardiac care assistance using a self-configured sensor network, as developed in [131]. Also, intelligent wireless sensors have been designed in [132] to perform data acquisition and limited processing. Individual sensor monitors specific physiological signals, communicates with other sensors and connects to a personal server. 

A couple of research studies [133,134,135] have adopted compressed ECG sensing as a promising approach to lower energy consumption in wireless body area networks for ECG monitoring. The wavelet representation of the ECG signal has been used to improve the performance of compression and reconstruction of ECG signals [133]. Earlier information about the wavelet dependencies across scales has been incorporated into the reconstruction algorithms and has exploited the high fraction of common support of the wavelet coefficients of consecutive ECG segments. Another research work [134] studied the energy efficiency of compressed sensing for ambulatory ECG monitoring. They propose a compressed sensing architecture, combining a redundancy removal scheme with quantization and Huffman entropy coding, to effectively improve the compression ratio. Comparably, in [135], a real-time energy-aware ECG monitoring system based on the emerging compressed sensing (CS) signal acquisition/compression paradigm for the WBSN applications has been proposed.

### 3.2. Technology-Aware ECG Monitoring Systems 

The second cluster of researches encompasses a group of monitoring systems that emphasizes the use of emerging technologies to support ECG monitoring. This group is classified into two categories: (1) the enabling technologies which involve IoT, Cloud, and Fog and (2) the monitoring devices which comprise mobile devices, wearable devices, and sensor devices. Table 3 depicts a selection of the most relevant work leveraging the new technologies involved in ECG monitoring systems.

#### 3.2.1. Enabling Technologies

ECG monitoring relies on key technologies to support various ECG processes, including preprocessing, processing, storage, analytics, and visualization of ECG signals. Cloud infrastructure provides storage and processing resources over the internet to support ECG monitoring systems. Fog computing is a very promising technology that brings the processing resources close to where the ECG data are generated, thus providing low latency and energy efficiency. IoT technology enables remote ECG monitoring using IoT devices to retrieve ECG signals, allowing transmission in real-time over the internet to the physician for further analysis.

Of particular interest are systems that propose solutions to optimize processing and reduce the cost of data transmission and storage over the Cloud infrastructure [24,26,71,143,144,145], this is also very crucial in cases of emergency and vital situations when real-time analytics are urgently required for actionable insights. Tuli et al. [26] incorporated ensemble deep learning in edge computing devices and deployed it for the real-life application of automatic heart disease analysis. Similarly, wearable devices were empowered with processing capabilities to locally (at the edge) analyze the signal and identify abnormal behaviors [144]. However, wearable embedded devices, mobile edge devices, and Cloud services were combined to provide reliable, accurate, and real-time heart monitoring [25,143]. Wearable devices are remotely trained to interpret heart abnormalities and the Fog extends the Cloud by migrating data-processing closer to the production site, thus accelerating the system’s responsiveness to events.

Other research initiatives have focused on the development of patient-centric monitoring solutions where patients play an active role in their health monitoring. While in the work of Xia et al. [71] a cloud-based system has been developed to assess the usefulness of the ECG data collected from patients themselves using either mobile devices or web applications. Wu et al. [29] addressed the comfort of monitored patients and designed a non-invasive textile electrode that guarantees excellent quality of ECG reading and offers comfort to patient while they are being monitored.

A great number of researches proposed relevant IoT-based solutions for ECG monitoring [12,27,132,136,137,138,139,140,141,142]; these researches are centered around the use of IoT devices for real-time ECG acquisition, processing, and analytics. The authors in [27,136,137], and [138] proposed an IoT-based patient-continuous monitoring system using the ECG sensor. All systems collect ECG data using sensors and process and analyze in real-time the collected ECG data. Subsequently, they allow remote access to the patient’s health condition, to third parties (e.g., doctor and nurse), in addition, an alert is sent in the case of a critical situation for timely intervention. Other solutions, such as in [132], go beyond the adoption of traditional IoT-based ECG monitoring systems and further incorporate intelligent wireless sensors within a personal area network to handle data acquisition and limited processing, which helped in improving monitoring efficiency and urgent reactions in cases of emergency. Similarly, in [12], nanomaterial-enabled ECG sensors were used, which improved conductivity, electrical proprieties, and reduced the induced monitoring cost. Moreover, in [141], Granados et al. proposed an IoT platform for real-time analysis and management of a network of bio-sensors and gateways. They explored the use of a Cloud deep neural network architecture for the classification of ECG data into multiple cardiovascular conditions.

More comprehensive work has been reported in [139], which proposed an interconnection framework for mobile health (mHealth) based on the IoT. The proposed framework details the hardware and software components along with the underlying network and protocol used, including 6LoWPAN and YOAPY protocols to support secure and scalable integration and deployment of sensors within the patient’s environment. The framework evaluates the system’s capability to provide continuous monitoring, ubiquitous connectivity, extended device integration, reliability, and security and privacy support.

#### 3.2.2. Monitoring Devices

A multitude of devices are being used nowadays for ECG monitoring systems. They are classified into mobile-based, wearable-based, and sensor-based devices.

Mobile-based devices for ECG monitoring involve a wide range of devices, including, for instance, smartphones, smartwatches, and pocket ECG monitors. Aljuaid et al. [146] evaluated the effectiveness of using a smartphone-based ECG monitoring device on the frequency of clinic visits of patients who experienced ablation of AF. This study proved that the use of smartphone-based ECG monitoring led to a significant reduction in patient visits to the clinic after the surgical intervention. Likewise, a mobile device was integrated with an Arduino microcontroller and various sensors, including an ECG sensor, to retrieve sensory data and display vital sign measurements and send notification messages and the user’s location to the healthcare provider if any abnormality was discovered [31]. Another piece of work reported in [32] combined an Arduino microcontroller with an Android-based smartphone to develop an intelligent healthcare system and provide elderly patients with medical services at home. The system integrates an artificial bee colony (ABC) algorithm for ECG R-peak detection. It is able to detect various abnormalities, such as high blood pressure, low blood pressure, fever, tachycardia and bradycardia; it also sends notifications in case of unexpected events.

Various research initiatives proposed solutions that integrate wearable devices within an ECG monitoring system [14,21,22,29,30,125,126,127,147]. These solutions are either integrated within an ambulatory setup, home environment, or patient/user setup and are used for the monitoring of various vital signs. While the authors in [21,125] proposed an ECG monitoring device for wireless monitoring of atrial arrhythmia, and Fung et al. [22] explored a wearable “on-body” ECG patch, which was unobtrusive, easy to use device, leading to increased device wear time and diagnosis yield. Another category of an ECG monitoring system involves innovative contactless sensors to retrieve ECG signals without disturbing the patient’s comfort. Rachim and Chung [126] proposed a system consisting of capacitive coupled electrodes embedded in an armband. The system integrates analysis features to detect a real-time heartbeat, and a filtering algorithm to filter distractions generated by body movement or other noises. Likewise, according to the authors of [147] a smartwatch was embedded with various built-in sensors such as an accelerometer, gyroscope and optical heart rate sensor and compared with a commercially available and clinically used non-contact sleep sensor. Also, in [14] a textile-based, contactless ECG monitoring system with sensors embedded in non-ICU environments was proposed. The system measures ECG signals non-obtrusively using capacitive sensors integrated within patients’ stretchers, beds, and wheelchairs. These contactless monitoring systems prove their robustness, and efficiency in recording quality signals and generate effective real-time analysis.

Most of the abovementioned ECG monitoring systems integrate sensing devices as a core component of the system to retrieve ECG signals and ultimately to do some light processing either at the edge or at the Cloud. Therefore, overlapping might be apparent with the other abovementioned categories, since they are embedding sensors within the ECG monitoring solution. Among these research initiatives include the subsequent propositions from [12,26,28,136,138,139,142]. For instance, Villarrubia et al. [142] proposed a monitoring and tracking system that uses virtual organization of agents for easy integration of different devices. It facilitates the integration of interactive television to moderate activity with the user under monitoring. Additionally, [148] proposes a button-like wearable wireless non-contact system for long-term multiple biopotential monitoring. It relies on ultra-high input impedance and is able to retrieve various biopotential signals, including ECG, through multiple layers of cloth without directly contacting human skin. This system proves its feasibility of extended monitoring without disturbing daily activities.

### 3.3. ECG Monitoring Systems Based on Schemes and Frequency

The third cluster of our experts’ classifications of ECG monitoring systems focuses on computational aspects. In this dimension, two subcategories are recognized. These are the monitoring scheme and the processing frequency. The monitoring scheme defines the spatial screening setup. ECG signals can be measured in stationary setups in different contexts while the patient is directly connected to screening and processing devices for diagnostic/prognostic purposes, which we refer to as a “Traditional Monitoring Setup”. Alternatively, ECG signals can be measured in mobile setups, where the patient is experiencing real-life activities for a full spectrum of monitoring purposes, such as activity monitoring, diagnosis, prognosis, or fitness monitoring, which we refer to as a “Real-time Monitoring Setup”.

In contrast to the monitoring scheme, the processing frequency defines the temporal screening setup. ECG signals can be processed in a constant setup that we refer to as “continuous monitoring,” a one-time setup that we refer to as “ad hoc monitoring,” or a recurring, prescheduled, and preplanned set up, which we refer to as “episodic monitoring.”

In this section, several ECG monitoring system research work are reviewed against the aforementioned criteria, highlighting technical complexities, sampling rates, advantages, and disadvantages of different approaches. Table 4 summarizes the research works reviewed in this section.

#### 3.3.1. Traditional ECG Monitoring

A number of research work addressed traditional ECG monitoring setups in various contexts such as hospitals [149,150,151], homes, or remote ambulatory settings [31,32,112,146,152]. Screening devices can be physically connected to processing devices [31,32,149,150], or wirelessly accessed [17,112,152,153].

Ad hoc (i.e., one-time), immotile monitoring has been deployed in many research studies to address various healthcare situations using different types of ECG sensors and monitoring platforms. For instance, Aljuaid et al. [146] used ECG Check (https://www.cardiacdesigns.com/) with smartphone technology to monitor AF patients post-ablation; Benini et al. [112] designed a single-lead sensor for home telemonitoring; Yousef and Hau [31] used an analog heart rate sensor in their mobile-based system (myVitalGear) for general health monitoring, and Rafiq et al. [152] designed a system with three-lead wet electrodes for home health monitoring. It was emphasized by a number of researchers that ad hoc, non-mobile monitoring can potentially lower the burden of outpatient clinical visits and AF-related visits in the post-ablation period. In their study, Aljuaid et al. [146] mentioned that traditional smartphone-based ad hoc home monitoring helped reduce the number of post-ablation patients’ visits by more than 50%.

However, in ad hoc monitoring settings, patients may forget to perform the monitoring tasks. In addition, for patients with critical health situations, it is vital to have regular recurrent screenings. To address these concerns, some research works proposed episodic monitoring that can be done at pre-scheduled intervals. For example, Hsieh and Len [32] incorporated an alarm function into the system to remind the patient to use the screening device. They pointed out that their monitoring system not only saves medical resources, but also enables elderly people to care for themselves, thereby promoting their health.

For critical and life-threatening cases in traditional setups, continuous monitoring is necessary, especially in ICUs. A number of research work focused mainly on neonatal patients, for instance, Bambang et al. [149] and Bouwstra et al. [150] who designed smart jacket-based continuous monitoring systems for prematurely born babies in the neonatal ICU (NICU). Alternatively, for elderly people who would be moving around, Ahmed et al. [17] utilized IoT-based technology for wireless sensor nodes positioned in the ICU and nursing rooms to provide continuous ECG monitoring of patients with severe cases and then transmit the signal to remote doctors. Precision in these situations is vital; therefore, a major challenge faced in ICU continuous and smart monitoring is the need to select a suitable filtering technique, since ECG signals are noisy, which need a suitable amplifying technique should be used, given that the ECG signal is measured in millivolts.

#### 3.3.2. Real-Time ECG Monitoring

In some cases, cardiologists may recommend 24-hour Holter monitoring for patient monitoring. A Holter monitor is a 12-lead medical device that records the heartbeat and checks for unusual signs. It is usually uncomfortable to apply the Holter 12-lead device to the patient’s body for 24 hours. Some attempts have improved the 24-hour Holter monitoring and utilized an adhesive patch that can be attached to the patient’s body, reducing the number of required leads in a typical Holter. For instance, Karaoğuz et al. [125] used a BeyondCare device applied to the upper left upper region of the subject’s chest beside the 12-lead Holter device, simultaneously, for palpitation assessment.

Recent advancements in wireless ECG monitoring systems have produced a wide variety of real-time monitoring systems, ranging from wearable textile-based monitoring systems, such as smart shirts [5] and textile electrodes [154,155,156], to contactless [157] ECG monitoring systems. In real-time setups, patients can measure ECG signals while doing normal real-life activities [161]. This has allowed for more prompt assessment and medical intervention when necessary. Furthermore, it substantially reduced the cost of healthcare expenses by lowering the number of hospital visits for traditional regular monitoring. For instance, Lee and Chung [5] designed a smart shirt that continuously measures ECG and acceleration signals remotely in real-time setup for health monitoring. Bianchi et al. [154] proposed the use of T-shirts and/or bed sheets with sensor electrodes to measure ECG signals and other vital signs to assess sleep and respiratory problems in real-life settings. Bsoul et al. [6] designed MedAssist, a continuous, real-time, single-lead, wireless monitoring system for the diagnosis of sleep apnea. On the other hand, the authors of [155,156] designed washable long-term wearable sensors for fitness and activity monitoring.

Despite the advantages of continuous monitoring in real-time setups, it generates an abundant amount of ECG signal data, which can give substantial signal noise and artifacts caused, sometimes, by abnormal physical activities, a problem that has been highlighted by many researchers [5,154]. This, in turn, emphasizes the need for noise filters and smart feature selection algorithms. Alternatively, episodic monitoring was adopted in several researches to limit the causes of motion artifacts and constrain the amount of generated ECG signal data, allowing for easier processing and analysis. For example, Yoon and Gho [157] utilized a commercial contactless ECG monitoring device for remote home telemedicine. In their experiments, they considered pre-planned, pre-scheduled three-time intervals focusing on the patient’s resting state and post-exercise state. Lee et al. [158] proposed a mobile cardiopulmonary rehabilitation system with a wireless ECG Holter to give real-time feedback during exercise in home-based environments. In contrast to pre-scheduled continuous monitoring, some researchers adopted signal-sampling techniques that can be used for event-based ECG monitoring. Signal sampling algorithms convert continuous-time signals into discrete-time signals. For example, Ravanshar et al. [159,160] implemented a level-crossing sampling technique to reduce the sampling rate from 1 kS/s to around 120 S/s. Consequently, a considerable reduction is achieved not only in the amount of processed signals but also in power consumption.

### 3.4. ECG Monitoring System Targets and Purposes

Several ECG monitoring systems in the literature have been developed to serve a certain purpose or to target a specific functionality, which we grouped into the fourth cluster. In this cluster, we classify ECG monitoring systems as service-based or performance-based systems. The service-based systems are focus on either medical or non-medical purposes. However, performance-based monitoring systems intend to concentrate on performance improvements. The following subsections detail the classification of several proposed ECG monitoring systems, along with examples of selected systems highlighting special key features and key research problems.

#### 3.4.1. Service-Based Monitoring Systems

In this section, we classify the service-based ECG monitoring systems into three main categories: diagnoses, activities, and prognoses. Numerous ECG monitoring systems have been developed for diagnosing specific or multiple diseases. Table 5 summarizes the aforementioned service-based ECG monitoring system classification. In Table 5, we further classify the systems focusing on disease diagnosis into two main categories: (1) general CVDs, having three subcategories including Arrhythmia, AF, and other abnormalities, such as, such as left ventricular hypertrophy [11]; unexplained syncope [162]; long QT syndrome [163]; depression [164] and coronary heart disease [165], and (2) sleep apnea.

In general, most of the systems use wireless and wearable devices for data acquisition and were designed for diagnosis purposes [166,167,168]. Other ECG monitoring systems mainly focus on the interpretation of diagnostic algorithms [88,166,169,170]. Arrhythmia diagnosis is one of the most common diseases diagnosed based on ECG signals [171,172,173,174]. Real-time Cloud-based ECG monitoring systems for arrhythmia detection were proposed in [175,176], whereas AF is commonly diagnosed based on ECG signals [57,67,177,178,179]. ECG signals contribute to the detection of several cardiovascular abnormalities, which were addressed in [180,181,182]. Developing evolutionary, efficient, and accurate automatic diagnostic techniques have been always an open area of research to overcome many challenges, among which are medical device capabilities, ECG diagnostics overlapping patterns, and other ECG signal-related issues throughout the system’s lifecycle.

Other systems support the objective of monitoring user activities; examples include sports, driving, daily activities, and elderly activities. ECG monitoring systems dedicated to sports activities represent a significant share among existing monitoring systems [37,38,183]. For example, Peritz et al. proposed a method for diagnosing athletes’ heart palpitations [184]. Additionally, a solution using multi-sensor wearable sports devices was proposed for heart rate detection under different subjects’ activity states, that is, different types of movements [185]. Another exercise evaluation platform has been developed by Sun et al. [186]. Furthermore, Pollonini et al. [187] proposed an integrated device for measuring oxygen transport during physical exercise based on ECG and PhotoPlethysmoGraphy (PPG) monitoring. The health of professional athletes, such as footballers was the main study in [188], where cardiovascular conditions and other health problems were evaluated for health risk assessment during their preretirement and postretirement years. Moreover, water sports monitoring was discussed in [36].

Other activities, such as driving, was tackled in the literature in [189,190,191,192,193,194,195]. These studies proposed ECG monitoring for the purpose of observing drivers’ health and emotions, with the ultimate goal of enhancing transport safety on the roads.

The elderly population was the objective of a good number of publications in the reviewed literature. Some of these researches study wearable devices for monitoring elderly activities [114,196], others propose the use of smartphones for monitoring [197], and forecasting short-term health conditions [198]. Daily activities using wearable monitoring devices were addressed in [33,34,35]. Furthermore, real-time activity monitoring at different premises, such as home, healthcare or sports facilities, was discussed in [155,156,199].

ECG systems related to activity monitoring tend to suffer from similar challenges faced by remote systems, among which are sensor characteristics, hardware constraints of on-board data processing, complexity and robustness of classification algorithms for mobility detection. Additionally, these systems have several ethical issues, including data collection (e.g., subjects’ privacy), as well as lack of monitoring system usage confidence (e.g., subject can manually manipulate the monitoring device). Prognosis is one of the main objectives of ECG monitoring systems attracting much research interest. This is because predicting the likelihood of expected disease development is considered a major human health concern nowadays. Many ECG monitoring systems were proposed not only to diagnose, but also to predict certain diseases such as arrhythmia [200,201], AF [63,202], Epilepsy [203,204], and other cardiovascular diseases [89,205,206,207,208,209,210,211,212]. Many studies that are related to daily monitoring and activities during sleep, proposed different designs and specifications for wearable devices, such as shirts [33,34,35].

Additionally, prediction based on ECG monitoring was addressed in various aspects of health status, such as survival chances, mood and behavior, and health status. Survival, risk of cardiac death and other predictions related to cardiac problems were discussed in [213,214,215]. Alternatively, the prediction of mood changes and response to depression treatment were addressed in [39,40,41]. General health status and activity prediction were proposed in [105,216]. An interesting study related to predicting heart motion to assist in robotic heart surgery was detailed in [217].

#### 3.4.2. Performance-Based Monitoring Systems

We define performance-based monitoring systems as those that address performance advances in different aspects and characteristics. In this section, we classify the performance-based systems into three main categories, considering the main factors affecting the performance, such as energy, cost and resource provision. Table 6 summarizes the researches reviewed in this section.

Energy conservation is one of the main contributors to better system performance and to enhance life quality. Several reviewed ECG monitoring systems proposed solutions for lower energy consumption by reducing signal transmission, processing, and supporting signal compression. Most of the studies promoted the use of Bluetooth Low Energy (BLE) for signal transmission to support low energy consumption [126,155,222,223,224,225,226,227,228,229,230,231,232]. Few other studies used alternative wireless methods to reduce transmission and eventually reduce power consumption [234,235]. However, the use of the aforementioned approaches implicate some challenges regarding the lack of interoperability of different wireless devices, short battery life, and some security problems. Another way of reducing energy consumption is optimizing processing techniques and proposing new enhanced algorithms for signal processing [222,233,236,237]. Alternatively, signal compression is used for lower energy consumption in [134,135,239,240,241,242,243].

In general, cost reduction is also an extremely important objective of many performance-based monitoring systems. Different approaches lead to cost reduction, including the use of low-cost devices, such as smartphones [232,244,245,246]. Other studies proposed special low-cost circuit designs [247]. Reducing the cost could also be achieved by using a low-cost wearable devices for signal acquisition, especially in IoT-based systems [248,249]. Other low-cost devices were used, such as portable ECG monitoring devices in [250,252]. Sanghavi et al. [10] proposed an IoT-based remote monitoring system, which includes a Raspberry Pi, Arduino Uno, ECG Sensor and an IoT Cloud for storing and plotting ECG data in real-time. A home-based monitoring system with low-cost data acquisition was proposed in [251]. Disease prevention is another perspective in reducing monitoring costs. This issue was addressed in several papers in the literature. Yong et al. [253] suggested prolonged ECG monitoring for secondary stroke prevention, which eventually eliminates the cost of stroke treatment consequences. Additionally, prolonged and active monitoring significantly improved the diagnosis of AF and was associated with significantly lower emergency department visits and hospitalization requirements [254].

Other resource-aware monitoring systems were proposed for efficiency improvement. For example, Wang et al. [255] proposed resource-aware Body Sensor Network (BSN) architecture for real-time healthcare monitoring. 

Management, processing, and storage of data in ECG monitoring systems introduce several challenges, one of which is the storage of an enormous volume of data efficiently while responding to dynamicity and scalability requirements. Storage and transmission improvements were proposed in a resource-efficient ECG monitoring system named Generative Model-Driven Resource Efficient ECG Monitoring (GeM-REM). In this system, the ECG data are stored as model parameters rather than data samples to reduce the storage space, and only the abnormal sensed ECG signals are transmitted to reduce energy [232]. Other resource allocation approaches were addressed in [222,233].

### 3.5. ECG Futuristic Monitoring Systems

The latest trends in ECG monitoring systems will revolutionize the way ECG signals are collected and processed to give valuable insights serving various purposes while protecting patients’ privacy and emotional health. Personalization and adaptation to various contexts as well as to various stakeholders will offer a new level of high-quality smart healthcare. Modern technologies will play a vital role in this radical transformation. These include radar cardiography, implants, robotics, steganography, and other AI technologies. Table 7 summarizes selected research works related to these five categories.

Continuous heart rate monitoring and immediate heartbeat detection are primary concerns in contemporary healthcare. Most of the research work reviewed in this study focuses on ECG signal monitoring that generally requires touch-based sensing of the patient’s skin. Patient skin irritation caused by touch-based sensory devices were highlighted by a number of researchers [97,125]. A convenient and reliable touch-free alternative is radar cardiography. Radar systems enable touchless, contactless, and continuous monitoring of heart rate through clothes, blankets, or other isolators. Plenty of the research work investigated the possibility of using radar systems for continuous heartbeat monitoring and detection of vital signs [256,257,258,259,260,261].

Alternatively, some researchers investigated implanted sensor technology for durable and long-term continuous monitoring [263,264]. For instance, Giancaterino et al. [262] utilized insertable cardiac monitors (ICMs) which are small, medically implanted devices to offer continuous ambulatory ECG monitoring with a lifespan of up to three years. Sunnet et al. [265] used an implantable loop recorder (ILR), which is a small devices with integrated leads that are implanted in a small subcutaneous pocket after a simple surgery, with a dedicated AF detection algorithm for long-term ECG monitoring in patients with atrial flutter. Implants offer a practical solution for long-term monitoring, given that continuous external monitoring for such a long period of time can be unfeasible.

On the other hand, advancements in robotics introduced new opportunities for cardiac healthcare, especially with the great challenge of limited medical resources. Some researchers investigated the dimensions of the practical use of robotic-assisted heart surgeries [217,266]. Others studied the possibility of having a robot assistant providing medical feedback, diagnosis, and notifications [267,268].

New AI technologies will extend ECG monitoring systems’ horizons beyond physiological disease assessment. ECG signal can be used to evaluate psychological state, emotions, and stress levels to aid mental healthcare for people living in stressful environments [59,269,270]. Advancement in deep learning technologies and algorithms can provide interesting opportunities for adaptation and personalization, overcoming individual differences by periodical retraining [271,272].

In using the aforementioned technologies for monitoring patients’ heartbeats and other vital signs, not only an enormous amount of ECG signal is collected, but also other physiological measurements, such as temperature, blood pressure, glucose levels, as well as patients’ personal data, are required. It is, therefore, utterly important that patients’ privacy is protected during data transfer over the communication networks, as well as being stored in hospital servers or used by remote monitoring systems. Several techniques and security protocols were proposed in the literature to protect users’ privacy. Techniques used can be categorized into two main classes. The first class of security techniques are focused on encryption and cryptographic algorithms [276,277]. These techniques are accused of having a large computational overhead, which makes them unsuitable in a resource-constrained mobile environment. The second class of security techniques is focused on concealing sensitive information inside another set of insensitive host data, with no increase in the host data size or computational overhead. These techniques are called steganography techniques. Steganography is defined as: “the art of hiding secret information inside another type of data called host data” [278]. A number of research work investigated steganography techniques to secure patients’ privacy during ECG signal collection, storage, and transmission [48,273,274,275].

## 4. Key Challenges of ECG Monitoring Systems

As discussed in this paper, ECG monitoring systems involve many components, variable contexts, and various stakeholders and encompasses diverse technologies. This diversity and variability of ECG monitoring system contexts and components impose a number of challenges that have been highlighted by several researchers. In the subsequent sections, we discuss ECG monitoring challenges related to the use of monitoring devices, signal quality, sensor design, durability, the size of the data, visualization, and integration.

### 4.1. Challenges Related to Usage of Monitoring Devices

Aljuaid et al. [146] pointed out that manual static screening (i.e., traditional monitoring) has a major limitation when carried out in home settings, since patients have to learn how to operate monitoring devices, such as ECG Check (https://www.cardiacdesigns.com/) or Kardia (https://www.alivecor.com/), as well as having sufficient knowledge in using heart monitoring smartphone applications, which sometimes may be a challenge for elderly and illiterate people. Hsieh and Len [32] highlighted that patients may forget to carry out monitoring tasks in ad hoc monitoring in home settings, reducing the gains sought from regular monitoring. Hence, alarm and screening reminder needs to be considered when designing home manual monitoring systems.

### 4.2. Challenges Related to Signal Quality

In real-time monitoring, patients can enjoy real-life activities, including physical exercise and running, which usually result in motion artifacts, signal noise, and deterioration. Lee and Chung [5] highlighted in their research the importance of combining efficient filtering methods for real-time monitoring setups with motion artifacts removal during running or physical exercise of a person. They proposed the use of an accelerometer as a source of noise reference.

High precision is very important in continuous ICU monitoring. However, the ECG signal is noisy and measured in millivolts, which accentuates the need for good filtering and amplifying techniques. This challenge was highlighted and addressed in [149,17].

### 4.3. Challenges Related to Monitoring Durability

For real-time monitoring, it is important to use energy-efficient devices and communication technologies to allow for long-term monitoring. This challenge was highlighted by many researchers [159,160,279]. Sampling algorithms were used by the authors of [159,160] to help conserving energy. Also, the authors of [279,146] used Bluetooth as a low-energy communication protocol. Furthermore, data dimensionality reduction techniques can be used to reduce data size, thus alleviating its processing, which will support monitoring durability.

### 4.4. Challenges Related to Size of ECG Signal Data

Real-time screening is usually conducted for a relatively longer period of time compared to traditional screening (e.g., days or even months). As a result, the amount of generated ECG signal data is usually large and sometimes massive. Subsequently, the process of signal analysis and interpretation turns into rather a challenging task. This accentuates the need for automatic analysis and interpretation of signal data for these monitoring setups in order to generate useful notifications for patients as well as health caregivers. Bianchi et al. [154] pointed out that for real-time remote monitoring setups, it is imperative to implement intelligent feature extraction algorithms to select informative time windows only from the signals and, eventually, transmit them to a remote station for interpretation.

### 4.5. Challenges Related to Electrode/Sensor Type and Design 

In addition to challenges related to the size and quality of ECG signal data, some challenges are related to electrode design, the number of leads, and the type of conductor used. For example, Fensli et al. [161] highlighted that the ECG signal recording differs in some ways depending on the type and number of electrodes used. It is, therefore, necessary to further explore the suitability of this recording principle for disease diagnostic purposes. Different diseases require different types of recordings, which should be supported by the selected electrode. Another design challenge is related to the patient’s comfort with sticky electrodes. Concerns around the adverse effect of electrodes on the human body were addressed in [97]. Moreover, Karaoğuz et al. [125] highlighted in their study that 7.3% of participants had minor skin irritations related to the sticky gel electrodes. Alternatively, the authors of [155,156] proposed a chemical formulation appropriate for the dipping of various textile fabrics (e.g., cotton, polyamide, and polyester), making flexible washable electrodes for long-term monitoring compared to gel electrodes, which are disposable and cause skin irritation. These textile washable electrodes also solved the common challenge known in gel electrodes related to the low signal quality when electrodes dry out. Nevertheless, challenges regarding the design of wearable devices require further research.

### 4.6. Challenges Related to Visualization

Gusev et al. [279] highlighted a few challenges in handling visualization for continuous ECG monitoring. Mainly, the problems were related to handling different refreshing rate requirements via multiple platforms installed on displaying devices with low processing requirements.

Long-term ECG data analysis can be challenging as it tends to oversimplify the visualized information, which results in losing significant components. Therefore, Jarchi et al. [280] proposed a visualization approach using classification for identifying heartbeats using long-term single-channel ECG.

Display adaptation and customization are another challenging issues for the ECG monitoring system. Display customization of data reporting for each stakeholder, such as the doctor, the nurse, the caregiver, and the patient, is included. Each stakeholder requires a different reporting context. Real-time display is also required to be automatically customized according to the visualizing device’s screen size and even the device battery level. These challenges were addressed in [281], in which special dashboard functionalities were integrated with visualization features, such as zoom-in and zoom-out, and filtering.

### 4.7. Challenges Related to System Integration 

Baig et al. [54] highlighted a few system integration challenges that face the existing clinical decision support models concerning scalability and reliability, pointing this out as a future research direction. They suggested exploiting Cloud resources for real-time processing to handle the integration issue. Furthermore, Jovanov et al. [282] proposed the seamless integration of information with sensors and other networks and the use of public resources and standard Internet methods for authentication and secure communication. Additionally, Spanò et al. [283] discussed opportunities for the seamless integration of remote monitoring systems with other smart home systems over an IoT infrastructure. Service-Oriented Architecture (SOA) has also been considered a very promising solution for integrating heterogeneous systems. This is the case for ECG monitoring systems where various technologies, data sources, and devices are used to collect, process, analyze, and visualize data over various interfaces.

### 4.8. Other Challenges

Other challenges related to ECG monitoring systems that are different from those mentioned earlier include those related to complex computational requirements, energy harvesting, and patient/user resistance to contributing to his/her monitoring using various technologies and sensors. The involvement of mobile devices in continuous ECG monitoring makes them less effective for computational, data-intensive processing. Though the mobile device improves some flexibility to the monitoring process, problems related to battery consumption and the limited processing capability of the device are still not resolved completely.

## 5. Discussion, Conclusion, and Future Direction

ECG monitoring systems have been studied thoroughly in the literature; however, the multi-dimensional aspects of these systems make it difficult for researchers, medical practitioners, and others to select, among these systems, those that fulfill their monitoring needs, match the context of their use, and support the required disease monitoring requirements.

In this paper, we carried out an extensive review of the literature related to ECG monitoring systems, focusing on different aspects including applicability, the technology used, architecture, lifecycle, classification, and challenges. We presented and discussed an expert-verified classification model. In our experts’ taxonomy, we decomposed ECG monitoring systems into context-aware ECG monitoring systems, technology-aware ECG monitoring systems, ECG monitoring systems based on schemes and frequency, ECG monitoring systems targets and purposes, and futuristic ECG monitoring systems.

Current development in ECG monitoring systems leveraged new technologies, such as deep learning, AI, Big Data and IoT to provide efficient, cost-efficient, fully connected, and powerful monitoring system. Enabling technologies provide huge opportunities for the advancement of ECG monitoring systems. IoT brings in remote, unconstrained connectivity and services that leverage data and facilitate timely, meaningful, and critical decisions for a better lifestyle. Furthermore, Fog processing and cloud processing contribute to an increased opportunity to improve efficiency and fulfill numerous in-demand scalable application services. Furthermore, blockchain technology enables security over a distributed environment for various transactions throughout the different layers of the ECG monitoring system architecture.

We shed light on this work on the ECG monitoring system’s lifecycle, which incorporates various processes that can be classified into primary processes and supporting processes. However, these processes are not distinctly defined in the literature; some overlap and others are better merged. None of the researches addressed the details of the complete lifecycle of an ECG monitoring system. In this paper, to the best of our knowledge, we tried to generalize a complete lifecycle, including all main processes starting from data acquisition, preprocessing, feature extraction, processing and, finally, visualization. We have also defined a set of supporting processes, such as signal selection, encryption, and compression, which are only required by specialized systems.

As a future direction, exploring the field of robotics and healthcare automation has the potential to transform the next generation of ECG monitoring systems and to simplify robotic-assisted surgery procedures, elderly care, and remote and in-hospital continuous patient monitoring. Robotic-assisted surgery should be performed with higher precision, control, and improved vision, paving the way for the revolutionary healthcare of tomorrow. Further future research directions include exploring the use of the fast-growing IoT and smart connected devices for preventive healthcare and supporting the detection of patients’ unusual medical problems or a change in behavioral patterns. Also, personalized monitoring systems should be raised to the next level in terms of being highly customized according to patients’ needs and interactive to allow special configurations and adaptations to users’ requirements for a better quality of life. Finally, another possible research direction is to add more intelligence to the patients’ surroundings, for example, embedding more sensors in the carpet to accurately detect patients’ movements in order to establish behavior patterns and detect any abnormalities, as suggested in [282].

To that end, we endorse that this work, with a detailed discussion on many relevant research work, provides a comprehensive state-of-the-art review of ECG monitoring systems. It can serve as reference for various researchers and stakeholders in the field to compare, understand, and value ECG monitoring system features. It also highlights the main challenges these systems exhibit in terms of adaptability, integration, monitoring quality and durability. Finally, it outlines a future vision of the next-generation ECG monitoring systems for healthcare.

## Figures and Tables

**Table 1:** Summary of the most common literature referring to ECG monitoring systems’ lifecycles, primary and supporting processes.

	Primary Processes					Supporting Processes				
	Signal Acquisition	Signal Preprocessing	Feature Extraction	Processing	Visualization	Signal Selection	Compression	Data Storage	Modeling	Encryption
[58]	✓	✓	✓	✓		✓				
[42,43,44,45,61,62,63]		✓		✓						
[64]				✓						
[46,47,65,66,67]		✓	✓	✓						
[68]		✓		✓					✓	
[62]		✓	✓	✓		✓				
[69]		✓		✓	✓					
[70]		✓		✓		✓				
[12]	✓			✓						
[13]		✓		✓				✓	✓	
[71]		✓		✓	✓			✓		
[72]	✓	✓		✓				✓		✓
[73,74]	✓	✓	✓	✓						
[61]	✓			✓						
[75]	✓	✓	✓	✓				✓	✓	
[76]	✓			✓				✓	✓	
[77]	✓	✓		✓						✓
[55]	✓	✓	✓	✓					✓	
[54]	✓			✓	✓			✓	✓	
[49]	✓		✓	✓	✓					
[78]	✓	✓		✓	✓			✓		
[79]		✓	✓	✓			✓			
[80,81,82]					✓					
[83]	✓	✓	✓	✓				✓	✓	
[84,85]		✓		✓			✓	✓		
[86,87]		✓	✓	✓					✓	
[88]	✓		✓	✓			✓			

**Table 2:** Classification of selected context-aware ECG monitoring systems.

Context-Aware ECG Monitoring Systems	Category	Selected Papers
**Home Setting**	**Telemonitoring**	[109,110,111,112]
	**Wearable continuous monitoring**	[18,19,20]
	**Elderly monitoring**	[24,113,114,115]
**Hospital Setting**	**ICU clinical setting**	[16,17]
	**non-ICU clinical settings**	[14,15,116,117]
	**Holter monitoring**	[36,118,119,120,121]
**Ambulatory Setting**	**Ambulatory cardiac/telemetry monitoring**	[8,21,22,23,122,123,124]
	**Wearable ECG monitoring**	[25,29,125,126,127,128]
**Remote Setting**	**Telemonitoring**	[10,129,130]
	**Smart device-based ECG monitoring**	[12,24,126,131,132]
	**Compressed ECG sensing**	[133,134,135]

**Table 3:** Classification of selected technology-aware ECG monitoring systems.

Technology-Aware ECG Monitoring Systems	Category	Selected Papers
**Enabling Technologies**	**IoT**	[12,25,26,27,132,136,137,138,139,140,141,142]
	**Cloud**	[24,26,143,144,145]
	**Fog/Edge**	[25,26,28,29]
**Monitoring Devices**	**Mobile-based**	[30,31,32,139,146]
	**Wearable-based**	[14,21,22,29,30,125,126,127,147]
	**Sensor-based**	[12,26,28,136,138,139,142]

**Table 4:** Classification of ECG monitoring systems based on scheme and frequency.

Computational Dimension		Monitoring Frequency	Selected Papers
**Monitoring Scheme**	**Traditional**	**Continuous**	(Smart Jacket [149,150]), (IoT [17])
		**Episodic**	(alarm-based [32])
		**Ad hoc**	(ECG Check [146]), (lab-designed single-lead [112]), (analog heart rate sensor [31]), (three-lead wet electrodes [152])
	**Real-time**	**Continuous**	(single lead EMP and 12-leads 24-h Holter [125]), (Smart Shirt [5]), (T-shirts/bed sheets with electrodes [154]), (single-lead wireless [6]), (wearable, washable, [155,156]),
		**Episodic**	(pre-scheduled assessment [157]), (pre-scheduled pulmonary rehabilitation [158]), (event-based [159,160]),

**Table 5:** Classification of selected service-based ECG monitoring systems.

Service-Based	Category		Selected Papers
**Diagnosis**	**Cardiovascular Diseases**	**Arrhythmia**	[56,171,172,173,174,175,176]
		**Atrial Fibrillation**	[57,67,177,178,179]
		**Other Abnormalities**	[11,88,162,163,164,165,166,167,168,169,170,180,181,182,218]
	**Sleep Apnea**		[6,219,220,221]
**Activities**	**Elderly**		[114,196,197,198]
	**Sports**		[36,37,38,183,184,185,186,187,188]
	**Drivers / Driving**		[189,190,191,192,193,194,195]
	**Daily Activities**		[33,34,35,155,156,199]
**Prognosis**	**Cardiovascular Diseases**	**Arrhythmia**	[200,201]
		**Atrial Fibrillation**	[63,202]
		**Other Abnormalities**	[89,205,206,207,208,209,210,211,212]
	**Epilepsy**		[203,204]	
	**Survival**		[213,214,215]
	**Mood Related**		[39,40,41]
	**Health status and activity prediction**		[105,216]

**Table 6:** Classification of selected performance-based ECG monitoring systems.

Performance-Based	Category		Selected papers
**Energy**	**Transmission**	**BLE**	[126,155,222,223,224,225,226,227,228,229,230,231,232,233]
		**Other Wireless**	[234,235]
	**Processing**		[222,223,236,237,238]
	**Compression**		[134,135,239,240,241,242,243]
**Cost**	**Device**	**Phone**	[231,244,245,246]
		**Circuit**	[247]
		**Wearable**	[248,249]
		**Others**	[10,250,251,252]
	**Disease Prevention**		[253,254]
**Resources**	**Storage**		[232]
	**Other resource allocation**		[222,233,255]

**Table 7:** Classification of selected futuristic ECG monitoring systems.

Futuristic Dimension	Selected Papers
**Radar Cardiography**	[256,257,258,259,260,261]
**Implants**	[262,263,264,265]
**Robotics**	[217,266,267,268]
**AI**	[59,269,270,271,272]
**Steganography**	[48,273,274,275]
